# Implementing a standardised perioperative nutrition care pathway in upper gastrointestinal cancer surgery: a mixed-methods analysis of implementation using the Consolidated Framework for Implementation Research

**DOI:** 10.1186/s12913-022-07466-9

**Published:** 2022-02-25

**Authors:** Irene Deftereos, Danielle Hitch, Sally Butzkueven, Vanessa Carter, Kate Fetterplace, Kate Fox, Aurora Ottaway, Kathryn Pierce, Belinda Steer, Jessie Varghese, Nicole Kiss, Justin M Yeung

**Affiliations:** 1 Department of Surgery, Western Precinct, Melbourne Medical School, The University of Melbourne, Melbourne, Australia; 2 Department of Nutrition and Dietetics, Western Health, Melbourne, Australia; 3 Allied Health, Western Health, Melbourne, Australia; 4 Occupational Therapy, Deakin University, Geelong, Australia; 5 Cancer Services, Western Health, Melbourne, Australia; 6 Department of Allied Health (Clinical Nutrition), The Royal Melbourne Hospital, Melbourne, Australia; 7 Department of Medicine and Radiology, Melbourne Medical School, The University of Melbourne, Royal Melbourne Hospital, Melbourne, Australia; 8 Department of Nutrition and Dietetics, St Vincent’s Hospital Melbourne, Melbourne, Australia; 9 Nutrition and Speech Pathology Department, Peter MacCallum Cancer Centre, Melbourne, Australia; 10 Institute for Physical Activity and Nutrition, Deakin University, Geelong, Australia; 11 Allied Health Research, Peter MacCallum Cancer Centre, Melbourne, Australia; 12 Western Health Chronic Disease Alliance, Western Health, Melbourne, Australia; 13 Department of Colorectal Surgery, Western Health, Melbourne, Australia; 14 Sunshine Hospital, Level 3, WCHRE Building, 176 Furlong Rd, St Albans, VIC 3021 Australia

**Keywords:** Gastrointestinal cancer, Nutrition support, Nutrition care pathway, Qualitative, Dietitian, Multidisciplinary team

## Abstract

**Background:**

Implementation studies of complex interventions such as nutrition care pathways are important to health services research, as they support translation of research into practice. There is limited research regarding implementation of a nutrition care pathway in an upper gastrointestinal (UGI) cancer population. The aim of this study was to comprehensively evaluate the implementation process of a perioperative nutrition care pathway in UGI cancer surgery using The Consolidated Framework for Implementation Research (CFIR).

**Methods:**

This was a mixed methods implementation study conducted during a pilot study of a standardised nutrition care pathway across four major hospitals between September 2018 to August 2019. Outcome measures included five focus groups among study dietitians (*n* = 4–8 per group), and quantitative satisfaction surveys from multi-disciplinary team (MDT) members (*n* = 14) and patients (*n* = 18). Focus group responses were analysed thematically using the CFIR constructs, which were used as a priori codes. Survey responses were summarised using means and standard deviations. A convergent parallel mixed methods approach according to CFIR domains and constructs was used to integrate qualitative and quantitative data.

**Results:**

Qualitative data demonstrated that dietitian perceptions primarily aligned with five CFIR constructs (networks and communications, structural characteristics, adaptability, compatibility and patient needs/resources), indicating a complex clinical and implementation environment. Challenges to implementation mostly related to adapting the pathway, and the compatibility of nutrition coordination to existing aspects of care within each setting. Identified benefits from dietitian qualitative data and MDT survey responses included increased engagement between the dietitian and MDT, and a more proactive approach to nutrition care. Patients were highly satisfied with the service, with the majority of survey items being rated highly (≥4 of a possible 5 points).

**Conclusions:**

The nutrition care pathway was perceived to be beneficial by key stakeholders. Based on the findings, sustainability and compliance to this model of care may be achieved with improved systems level coordination and communication.

## Introduction

Malnutrition is highly prevalent in patients with upper gastrointestinal (UGI) cancer and is associated with poor patient outcomes [1]. Preoperative nutrition intervention is recommended for patients undergoing surgery for UGI cancer [2, 3], however high-quality evidence around implementation of recommendations into practice is lacking [4].

One approach utilised to implement nutrition support in oncology cohorts is a nutrition care pathway. Care pathways are complex interventions that support shared decision making and care provision for in specified patient groups over a defined period of time, for the purpose of improving patient outcomes, promoting safety and satisfaction, and optimizing resource allocation [5]. Several studies have identified benefits on nutritional and clinical outcomes in oncology populations, including improvements in access to care, nutritional status, weight and oncological treatment tolerance [6]. However no studies have evaluated the application of a nutrition care pathway in an UGI surgical oncology population.

Complex interventions require evaluation of implementation in health services research, as they support uptake of new evidence that improves care effectiveness and quality, particularly for interventions implemented across varied health services or settings [7]. The use of nutrition care pathways is an appropriate target for implementation research, as a means of understanding key contributing factors leading to implementation success related to process, mechanisms and contextual factors [7]. The aim of this study was therefore to provide a detailed mixed methods analysis of the process of implementing a standardised nutrition care pathway for UGI cancer surgery into clinical practice from the perspectives of dietitians, multi-disciplinary team (MDT) members and patients using a validated theoretical framework, The Consolidated Framework for Implementation Research (CFIR) [8].

## Methods

### Standardised nutrition care pathway and implementation

The standardised nutrition care pathways was developed and implemented across four major metropolitan hospitals in Melbourne, Australia, in a prospective pilot study with historical controls. The study commenced in September 2018, with 6 months of recruitment and a 6 month follow up period. Patients who were ≥ 18 years and planned for curative intent surgery for oesophageal, gastric or pancreatic cancer (*n* = 35) participated in the intervention group, after providing written consent. Implementation of the pathway was performed by a study lead dietitian at each site (*n* = 4), with a total of 12 dietitians across the four sites assisting with day-to-day implementation, each with at least 6 years of clinical experience. The pathway was developed through literature review of existing evidence-based guidelines and expert consensus of surgical oncology dietitians at all participating sites. The pathway included guidelines for the timing, frequency and type of dietetics intervention patients should receive based on nutrition risk stratification and was aligned with key perioperative treatment timepoints (diagnosis/planning, neoadjuvant therapy, pre surgery and surgery). The nutrition care pathway was delivered in the context of a multi-disciplinary setting. Dietitians attended the weekly surgical oncology multi-disciplinary meeting and self-referred patients into the pathway. The key aspect of the model of care allowing implementation of the pathway was the initiation of a preoperative dietetics outpatient clinic, which was co-located within the weekly surgical oncology clinic. Further details of the nutrition care pathway, study sites and participants, pathway development and implementation, and the levels of evidence underpinning recommendations is to be published in an additional manuscript.

**Fig. 1:**
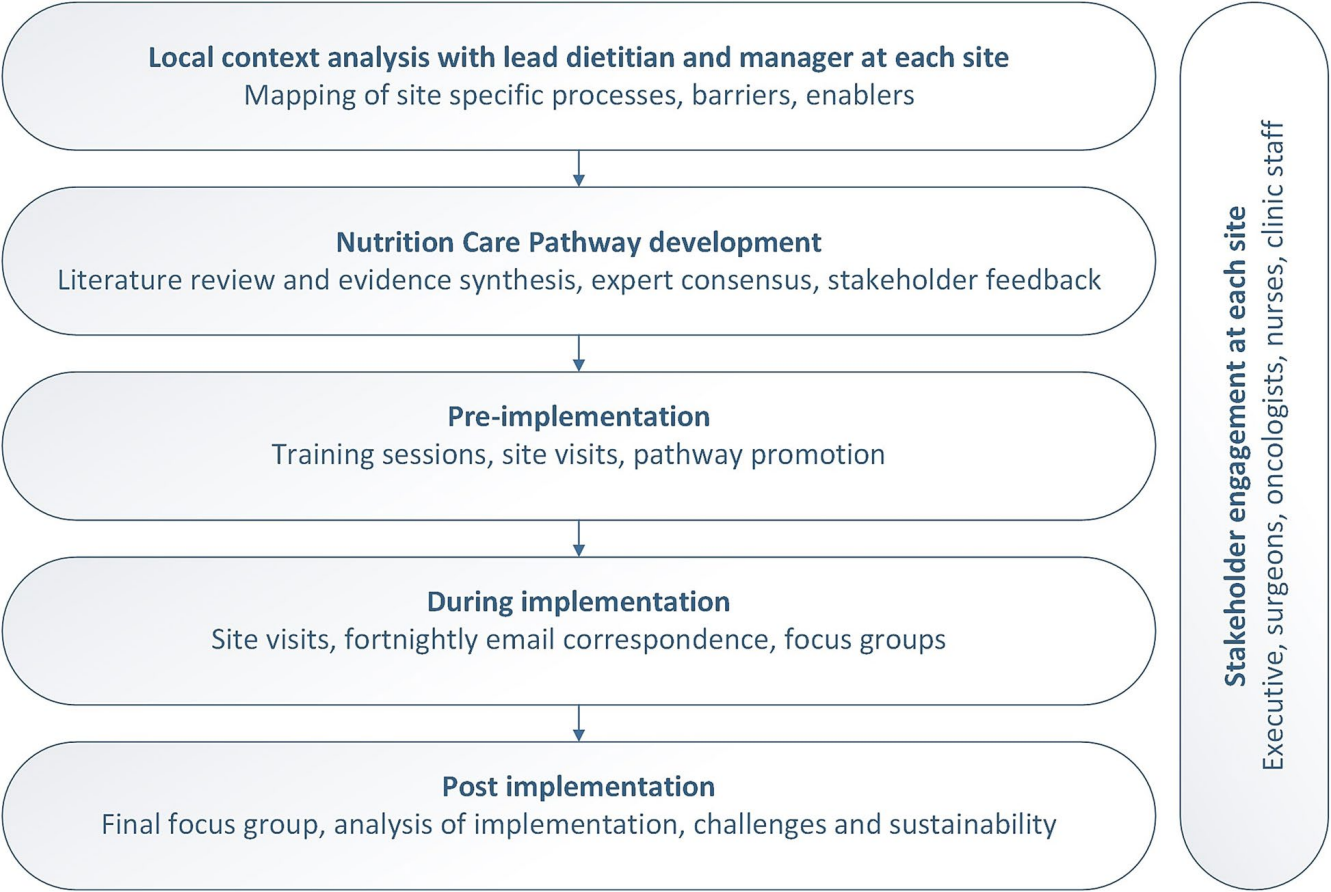
Structured implementation process

A structured approach to implementation was utilised, as outlined in Fig. 1. The implementation process was led by a dietitian with prior experience in development of a nutrition pathway in UGI cancer. Prior to implementation, training was provided to site dietitians by the lead dietitian, and site visits were also conducted before and mid-way through the project. The pathwas was promoted at each site through communication with key stakeholder groups (dietetics, surgical, nursing, executive and clinic staff) via emails and presentations at team meetings. Posters were also developed for patients and staff and placed at key clinic locations.

Focus groups with site dietitians were conducted during the pilot period (see section below) to discuss barriers and enablers to implementation.

### Theoretical framework

The CFIR was utilised to describe and evaluate the nutrition care pathway implementation process for this study. The CFIR aims to determine ‘what works, where and why’, [8] and it comprises five domains (Table 1) incorporating 39 constructs. These domains and components interact at multiple levels, and are interdependent [8]. The CFIR provides a rigorous structure for the principles of process evaluation, as previously described by Proctor [9] and Moore [7], and enables comprehensive evaluation of implementation by exploring the relationship between domains, constructs and outcomes [8, 10]. While the CFIR’s complexity can pose challenges to methodological design, it is valid to select the constructs most relevant to the study topic [10].

**Table 1:** Summary of findings according to the Consolidated Framework for Implementation Research constructs and representative quotes from dietitian focus groups

CFIR domain and construct themes	Summary of findings	Additional representative quotes from study dietitians (*n* = 4–8) who participated in the focus groups
**1. Intervention Characteristics:** features or characteristics of intervention.		
1.1 Adaptability	Dietitians needed to adapt the pathway due to individual patient variability (particularly at diagnosis).	“One of the challenging aspects is the screening part of it, and to really pin down the story behind it.” [December 2018] “As we have experienced, it can be really quite fluid and the plans at the beginning can sort of take a little bit of time to really solidify. So, keeping track of all of that, particularly when it’s going over multiple weeks.” [August 2019]
1.2 Complexity	Complex clinical situations were reflected by complexity within the NCP, which is directly linked to ongoing clinical care.	“It’s quite a complex, you know, following the pathway with multiple different time points…” [November 2018] “I think you just really learn a lot in terms on how complex the decision making is around these people.” [August 2019]
1.3 Relative advantage	Co-location of the dietitian in clinic was more advantageous to patient outcomes and MDT relationships.	“I think it’s been a great way to better communicate with the surgeons and touch base with patients more easily now.” [December 2018] “If you were sitting in hospital before your surgery, you were inpatient, you didn’t get seen by a dietitian beforehand. …now if you’re an outpatient, you will get much more proactive care.” [August 2019]
**2. Outer Setting:** features or characteristics of the external context or environment.		
2.1 Patient needs and resources	The outpatient model of care met patient needs by providing timely dietetic care, while balancing their emotional needs was difficult. Adaptations to the NCP increased care coordination time.	“It’s exhausting by the end of it because you have to wait for them to see the doctors and for us it’s surgeon and oncologist at the same time. It gets quite overwhelming.” [November 2018] “I was able to spread the assessment and then recruitment over 2 weeks in effect.” [November 2018] “He couldn’t come in on a weekly basis just to see me, so we’ve been doing some phone reviews.” [December 2018] “I know the patient… is really engaged…[and she’s] really taking on the advice that I’ve given her.” [December 2018] “We consider a weekly phone call. We’ve offered that and none of the patients are interested in that. They just want to see us when they come to their chemo and they’re sitting in the chair and doing it face to face.” [February 2019] “And there’s literally no way of knowing if the patient even knows about the diagnosis yet, so it makes it quite difficult for then that dietitian to get onto them early.” [February 2019] “They need to process the information about the diagnosis first, and then engaging with them on the nutrition level is better to come a little bit later down the track once they’ve had a little bit of time to process it all.” [August 2019] “I think in a way you become one of the patient’s main contact points for like a gateway to the team … they really valued being able to get in contact whenever they felt they needed to.” [August 2019]
**3. Inner Setting:** features or characteristics of the implementing organization.		
3.1 Networks and communication	Communications regarding medical treatment plans and appointments remained a consistent barrier to effective NCP implementation. Communication with the surgical oncology team around nutrition was perceived to significantly improve throughout implementation.	“Like whereabouts are they in the treatment pathway and making sure that nobody slips through the cracks. That, I have found, takes a bit of time as your numbers are bigger.” [November 2018] “And you think, okay, they are coming Thursday, I’m going to see them, but they’ve already come on the Monday.” [January 2019] “Patient discussed [in the MDM], nothing on the system and can’t figure out where he is up to…. things aren’t documented. There are lots of offline discussions that are not documented.” [January 2019] “So a lot of checking and checking and checking and checking to make sure that we actually catch them.” [January 2019] “Because she [the nurse coordinator] was able to just tell us…they come in next week and I’ll make sure that they see you and things like that.” [November 2018] “I think it’s been a great way to better communication with the surgeons and touch base with patients more easily now…it’s great.” [December 2018] “Surgeons are now coming and knocking on the door more than they ever have before.” [December 2018]
3.2 Structural characteristics	Structural characteristics of health services (e.g. shared care, rural and regional patients) created significant implementation barriers. Sites in a single campus or location reported less structural barriers.	“The handing them back and forth between oncology and then back to us and then, you know, have they been booked into clinic yet? Have they been discussed the MDM yet?” [November 2018]“I want to see them if they’ve got surgery in 2 weeks. So they definitely want them to come in if they are within the vicinity. I guess with rural patients, it’s been trying to call them and leave a message.” [December 2018] “It hasn’t been straight forward… the dietitian has had to chase them if they haven’t been booked back into clinic. So, staying on top of where they are at is a bit tricky…almost have to take on the role of coordinator.” [January 2019]
3.3 Compatibility	A weekly clinic model was most compatible with NCP; however workflows could not always align with the NCP.	“Say if they’re coming in ad hoc, if their preadmission clinic appointment was a different day; that’s the first time I’m actually seeing them face to face.” [November 2018] “He was coming for clinic that day, and he was right there. It was a really good time to have a chat with him. He sounded like he really needed some dietetic intervention.” [December 2018] “I’ve spent maybe two and a half hours screening the patient to figure out, then calling [the medical team] and asking, then calling [the nurse], and trying to really map out what’s been happening to the patient.” [December 2018] “It’s just sort of balancing sometimes the operations, you’ve got students, or you’re covering.” [August 2019]
3.4 Available resources	Insufficient resources allocated because time required to coordinate care was underestimated. The weekly clinic quickly began to exceed capacity.	“I find that if I had one person [like a coordinator] to go to that could tell me the answers, that would save me from emailing (NAME).” [December 2018] “A lot of patients are being seen late in the clinic. I am struggling to catch them before they go.” [January 2019] “Given the scope of how serious some of these cancers are and the timeframes from time of diagnosis to surgery, I feel as though I need an assistant to help me with some of [the coordination].” [February 2019] “In that clinic, the dietitian will be trying to see all of the preop patients, and the newly diagnosed patients as well as all about postop follow-up patients within the same clinic. And it can really get quite busy.” [August 2019]
**4. Individual characteristics:** characteristics of individuals involved in implementation.		
4.1, 4.2 Knowledge and beliefs around the intervention, and Self-Efficacy	Evidence limitations were well recognised and understood by dietitians. They used clinical reasoning to adapt the pathway and meet any challenges. Despite the gaps in the evidence, the NCP was also perceived as supportive of the significant role dietitians can play preoperatively and consolidate understanding of the patient treatment journey.	“But unfortunately, there’s just not enough evidence to say that everyone should get [a feeding tube].” [November 2018] “It solidifies my understanding of the whole pre surgical treatment, how that all works in the hospital. That has been a massive positive.” [January 2019] “So it does come down to clinical judgement and discussions with the team about things like feeding tube insertions…But I think it can be quite overwhelming to want to follow it specifically.” [August 2019] “But what we noticed was situations when we were actually using clinical judgment… making that decision was actually okay.” [August 2019]
**5. Process:** implementation strategies or tactics.		
5.1 Engagement	Engagement between dietitians and other members of the MDT was observed to improve throughout the study.	“The [clinic nurses] are more open to helping us to identify patients, they have a better understanding of how the patients will get through their treatment, what to expect...and the patients appreciate the support so much.” [August 2019]
5.2 Execution	NCP perceived to be moderately acceptable, feasible and implementable.	“I have found that it can be a bit of a challenge staying on top of things, as your numbers grow and grow [to keep track of] where patients are at in their treatment pathway.” [November 2018] “I was finding sometimes that the clinic would blow out a little bit and the patients themselves might start to get a little bit restless and probably just completely overloaded with information.” [August 2019]
5.3 Reflection	Dietitians highlighted the positive aspects of implementation (i.e. providing more proactive care, improved MDT relationships, greater understanding of the patient journey). The main implementation barrier was the complexity and variation in patient care.	“I think I didn’t anticipate as much coordinating of those sorts of things, I thought it’d be a bit more streamlined, but I guess, I got a really good realistic perspective of it.” [February 2019] “Knowing each patient’s journey is so different – a very heterogenous group even within the same tumour stream.” [January 2019] “That’s really, really encouraging to say that all the hard work resulted in some improved care, more proactive care and changes being seen.” [August 2019]

*CFIR* Consolidated Framework for Implementation Research, *MDM* Multi-Disciplinary Meeting, *MDT* Multi-Disciplinary Team, *NCP* Nutrition Care Pathway

### Qualitative focus groups

Focus groups with site study dietitians (including the four site leads) were conducted during the recruitment period, and at conclusion of implementation, in order to reflect the emerging aspects of implementation as the study progressed. There were four focus groups conducted monthly from November 2018 (2 months after study commencement) to February 2019. As no new themes or information were being generated, focus groups were then ceased and a final focus group was conducted at the end of recruitment to ensure no new themes were subsequently generated (August 2019). Attendance at each focus group varied based on how many clinicians at each site were implementing the pathway at the time (*n* = 4–8). The focus groups (average length of time 60 min) were run by the lead researcher (a dietitian), and were based on a semi-structured question guide developed to facilitate discussion of implementation, including barriers, enablers, successes and challenges. The focus groups were audio-recorded and transcribed verbatim. The focus group question guide is provided as a supplementary file.

### Quantitative surveys

Purpose-built surveys were developed for surgical, oncology and nursing stakeholders as no suitable surveys were available. These were tested by members of the project team prior to utilisation. The mixed methods staff survey evaluated perceptions and satisfaction with the pathway across 14 questions. Quantitative questions utilised five-point Likert items, while qualitative questions sought elaboration on service provision, patient outcomes and areas for improvement. These surveys were distributed in the MDT clinic and meetings at each site at the conclusion of the study period, and collected in hard copy by the project officer, to limit the potential bias if collected by dietitians, and maintain anonymity. Participation rate and discipline group (surgical or oncology) were unable to be determined as surveys were not distributed individually, in order to retain anonymity due to the small number of staff at each site. Patients who participated in the intervention group of the pilot study (*n* = 23) received the modified Patient Satisfaction with Clinical Nutrition Services (PSCNS) questionnaire, which has been validated in cancer outpatients [11]. The survey includes 19 questions on a five-point Likert scale regarding staff presentation and interpersonal skills, perceived health benefit, written information and fulfilled expectations. These anonymous surveys were distributed and collected by nursing staff in hard copy. The control group could not be surveyed as this group consisted of historical controls.

### Statistical analyses

Initial data analysis utilised a qualitative approach, with all data collected assessed for its alignment to CFIR domains and constructs, by coding at the question or variable level. The constructs were used as a priori codes, with a code book developed from Damschroder et al.’s definitions [8] to enhance analytical rigour [12]. These codes were also applied to focus group data, which were independently coded by two researchers (ID, DH), with disagreements resolved by discussion. Data within each CFIR domain was then analysed according to its original form. Qualitative data was evaluated with thematic analysis, utilising the previously developed codebook. Quantitative data were analysed descriptively, using counts and percentages, and compared with the qualitative findings. A convergent parallel mixed methods approach according to CFIR domains and constructs was used, in order to triangulate findings and seek complementarity between quantitative and qualitative data [13]. The final analysis presents the integrated findings by construct and domain.

The study is reported according to the Standards for Reporting Implementation Studies (StaRI) checklist [14], and the Good Reporting of A Mixed Method Study (GRAMMS) guideline [15]. Ethics approval was obtained from the Human Research Ethics Committee in June 2018 (HREC/18/MH/90), with site governance secured prior to commencement.

## Results

### Qualitative focus groups

Table 1 provides a summary of the integrated analysis of dietitian focus group data (*n* = 5 focus groups, 4–8 dietitians per session, 33–67% sampling rate, with at least one dietitian representative from each site), arranged around the CFIR construct themes. The CFIR constructs of ‘networks and communication’ and ‘structural characteristics’ were most frequently discussed by participants.

### Quantitative surveys

Fourteen members of the MDT completed the purpose-built satisfaction survey. The 23 patients who participated in the intervention received the PSCNS survey, and 18 (78% response rate) completed the survey. Tables 2 and 3 outline the results of the MDT and patient surveys, which were included in the integrated analysis of CFIR construct themes.

**Table 2:** Results of the purpose built multi-disciplinary team satisfaction survey

Survey item (rating from 1 to 5)^a^	Scores out of 5 N (%) participants	CFIR construct theme	Additional free text comments
Patients have access to adequate dietetic intervention prior to their surgery to optimise them for surgery.	2 = 1 (7.1%) 3 = 2 (14.3%) 4 = 6 (42.9%) 5 = 5 (35.7%)	3.3 Available Resources	“[Would like] more dedicated dietitian time.” “[Would like] better resourcing.”
The dietitian sees patients at the right times prior to their surgery	3 = 1 (7.1%) 4 = 8 (57.1%) 5 = 5 (35.7%)	2.1 Patient needs and resources	“Flagging of high-risk patients.”
There is a clear process to ensure that dietitians know about all patients undergoing curative Upper GI surgery prior to their inpatient admission	2 = 1 (7.1%) 3 = 6 (42.9%) 4 = 1 (7.1%) 5 = 6 (42.9%)	3.2 Structural Characteristics	
Patient oncology/surgical and nutritional care is well coordinated during all phases of the patient treatment from diagnosis/planning stage to time of surgery	2 = 1 (7.1%) 3 = 3 (21.4%) 4 = 7 (50.0%) 5 = 3 (21.4%)	3.1 Networks and communication 3.2 Structural Characteristics	
There is good communication between the oncology/surgical team and the dietitians about individual patient care during all phases of the patient treatment from diagnosis to discharge.	2 = 1 (7.1%) 3 = 1 (7.1%) 4 = 4 (28.6%) 5 = 8 (57.1%)	3.1 Networks and communication	“Better interactions, easier to refer [patients].”
Overall, I am satisfied with the level of nutritional care that patients are receiving in the pre-operative period	2 = 1 (7.1%) 4 = 7 (50.0%) 5 = 6 (42.9%)	5.1 Engagement 1.3 Relative advantage	
Patients appear satisfied with the input they receive about their nutrition. In the preoperative period	2 = 1 (7.1%) 3 = 3 (21.4%) 4 = 5 (35.7%) 5 = 5 (35.7%)	2.1 Patient Needs and Resources	“Better outcomes, patients are happy” “patients often remarked on dietitian’s advice positively.”
There are benefits for all patients undergoing curative Upper GI surgery to see the dietitian prior to surgery.	4 = 4 (28.6%) 5 = 10 (71.4%)	5.1 Engagement	
Only high-risk patients should see the dietitian prior to surgery.	1 = 4 (28.6%) 2 = 6 (42.9%) 3 = 1 (7.1%) 4 = 2 (14.3%) 5 = 1 (7.1%)	5.1 Engagement	
I believe there are improvements that can be made with the dietetic care that patients receive in the pre-operative period.	2 = 2 (14.3%) 3 = 3 (21.4%) 4 = 7 (50.0%) 5 = 2 (14.3%)	1.3 Relative advantage	
I believe that increased dietetic care for patients pre-surgery may lead to improved surgical and nutritional outcomes	3 = 3 (21.4%) 4 = 2 (14.3%) 5 = 9 (64.3%)	1.3 Relative advantage	
Overall, the nutritional care under the Nutrition Care Pathway is improved compared to the previous model	3 = 6 (42.9%) 4 = 2 (14.3%) 5 = 6 (42.9%)	1.3 Relative advantage	“Better availability.” “Increased preoperative involvement.” “Positive outcomes in patient care.”

^a^Rating for all items was 1 = strongly disagree to 5 = strongly agree

**Table 3:** Results of the Patient Satisfaction with Clinical Nutrition Services survey

Survey Item (rating from 1 to 5)^a^	Score 3/5 N (%) participants	Score 4/5 N (%) participants	Score 5/5 N (%) participants
**Perceived health benefits**			
1. The care I received from the dietitian has improved my general health	3 (16.7)	8 (44.4)	7 (38.9)
2. The care I received from the dietitian has improved the results of my medical treatment	3 (16.7)	7 (38.9)	8 (44.4)
3. The care I received from the dietitian has helped me to recovery faster	4 (22.2)	7 (38.9)	7 (38.9)
4. The care I received from the dietitian has helped my body to heal	3 (16.7)	7 (38.9)	8 (44.4)
**Staff presentation and interpersonal skill**			
5. The dietitian listened carefully to what I had to say	1 (5.6)	5 (27.8)	12 (66.7)
6. The dietitian was attentive to my needs	0 (0)	6 (33.3)	12 (66.7)
7. The dietitian came up with a good plan for helping me	0 (0)	8 (44.4)	10 (55.6)
8. The dietitian was well presented	1 (5.6)	5 (27.8)	12 (66.7)
9. The dietitian was polite and courteous	0 (0)	5 (27.8)	13 (72.2)
10. The dietitian was friendly	0 (0)	6 (33.3)	12 (66.7)
**Fulfilled expectations**			
11. The nutrition care I received was helpful	0 (0)	10 (55.6)	8 (44.4)
12. The nutrition care I received met my expectations	2 (11.1)	7 (38.9)	9 (50.0)
13. I would recommend the nutrition service to other members of the community	1 (5.6)	6 (33.3)	11 (61.1)
**Written materials**			
14. The written materials were of a high standard	2 (11.1)	8 (44.4)	8 (44.4)
15. I found the written information very easy to understand	2 (11.1)	8 (44.4)	8 (44.4)
16. The written information was easy to read	3 (16.7)	7 (38.9)	8 (44.4)
17. The written information made sense	1 (5.6)	9 (50.0)	8 (44.4)
18. The written information was well presented	1 (5.6)	9 (50.0)	8 (44.4)
**Overall service**			
19. Overall, the nutrition service was	1 (5.6)	6 (33.3)	11 (61.1)

^a^Rating for items 1–18 was 1 = strongly disagree to 5 = strongly agree. Rating for item 19 was 1 = very poor to 5 = very good

### Integrated analysis of CFIR themes

#### Intervention characteristics

##### Adaptability

The dietitians’ ability to adapt their approach to care was identified as important to their experience of the pathway, given individual variability in patient entry points, timing and interventions. Dietitians quickly realized that not all patients follow a typical pathway, and this was particularly evident when patients were yet to be informed of their diagnosis or treatment plan. The need for ongoing adaptation to the patient’s situation was problematic for dietitians, given the self-referral nature of the pathway. Further examples of dietitians adapting the pathway are discussed in ‘patient needs and resources’.

##### Complexity

Dietitians also recognized the impact of the complexity associated with decision making and the treatment journey of these patients throughout the study period. This was particularly highlighted at the end of the study, with reflections on specific patient experiences.

##### Relative advantage

Dietitians highlighted improved patient and team relationships, facilitated by co-location of the dietitian in the clinic, as a particular advantage of the pathway in comparison to the standard model of care. A more proactive approach, and the use of an outpatient model of care were identified as comparatively positive aspects of the pathway by dietitians and MDT members.

#### Outer setting

##### Patient needs and resources

Dietitians had to consider the sensitive nature of the diagnosis and emotional wellbeing of the patient, and often waited until the initial surgical and oncology consultations were completed before seeing the patient in the clinic. Often dietitians saw patients late in the session, and patients were tired. Therefore, dietitians adopted other strategies to ensure patients did not become too overwhelmed. A key tension that emerged during the study was the notion of ‘wanting to intervene as soon as possible’ (as per the pathway), but ‘not wanting to upset the patient’ if their diagnosis or treatment plan remained unclear; which was more common for atypical patients. Dietitians also become aware of the need to be flexible with regards to scheduling follow up appointments according to the pathway. However, most patients preferred to be seen on the same day as medical appointments or treatments, and dietitians recognised that linking in with existing appointments where possible was more patient centred and effective than phone or dietitian only reviews. As patients developed a relationship with the dietitians throughout their treatment, the dietitians often became their key liaison person. The development in their therapeutic relationship may have influenced their engagement, and dietitians noticed patients becoming more proactive as they received further intervention. Results from the patient PSCNS survey indicated that the dietetics service was highly rated both overall, and in each section (perceived health benefits, staff presentation and interpersonal skills, expectations and written materials) (Table 3). Responses from the MDT survey also confirmed these findings (Table 2).

#### Inner setting

##### Networks and communication

The most discussed construct was ‘networks and communication’, with both positive and negative aspects reported by dietitians. Communication and engagement with the MDT were perceived to significantly improve throughout the study by both dietitians and MDT members, particularly as a result of dietitian presence at the multi-disciplinary meeting and weekly clinic. However, communication regarding patients’ medical treatment and appointments remained an ongoing barrier to effective implementation. Dietitians found it time consuming to navigate the patient journey in order to self-refer patients into the pathway or conduct follow ups, as changes in appointment schedules were not communicated with the dietitians. Communication barriers specifically included conversations regarding treatment plans occurring outside the weekly multi-disciplinary meeting and not documented in patients’ medical records, or patients receiving private follow up appointments. This created a significant ‘coordination burden’ for dietitians as the pathway was designed around patients’ pre-existing medical appointments and key stages in their treatment. No single person was responsible for ownership of communicating the treatment journey to the team. These issues were noticed particularly for patients who were from rural locations, were receiving shared care between organisations, or who required further tests to confirm diagnosis. Facilitators to implementation included communication and documentation of treatment plan within the weekly multi-disciplinary meeting, as often the appointment dates were arranged during that time. Having a nurse coordinator was also regarded as a significant advantage at one site.

##### Structural characteristics

Structural characteristics was the second most prevalent construct discussed by dietitians. Shared care between health services emerged as an important barrier to efficient and timely dietetics care. Sites that provided both surgical and oncology treatments onsite reported less concerns regarding this. A frequently encountered example of this issue was the logistical issues of booking patient appointments if they were not attending the allocated clinic, or if they were being managed by multiple clinicians or health services (particularly at the diagnosis stage when determining if patients were eligible to enter the pathway). Although, the physical co-location of the dietitian was beneficial for seeing patients and optimising management together with the medical team. The scores from the MDT survey also reflected some barriers around referral and entry into the pathway (Table 2).

##### Compatibility of workflows

Within the first 2 months of the study, it became clear that the pathway was most compatible with the weekly outpatient clinic model of care for which it was originally designed. The pathway was less compatible when patient care was delivered using different workflows (e.g. via phonecalls) or patients did not attend the surgical oncology clinic to see the medical team. In these cases, a significant amount of dietitian time was spent attempting to make the pathway compatible with the different workflow. Throughout the study, the dietitians’ other competing workflows (for example, staff leave) also impacted on the ability to run the pathway on a day-to-day basis.

##### Available resources

As more people joined the pathway, the level of coordination required due to the barriers described began to use a significant amount of time. However, the dietitians understood the importance of persisting with the pathway. Similarly, in the clinic, patient demand started to exceed resources within the first 3 months of implementation, and it was clear that allocated resources were insufficient. Members of the MDT also reflected that increased resources were required, however their feedback on the adequacy of patient access to dietetic intervention prior to surgery was positive overall (Table 2).

#### Individual characteristics

##### Knowledge and beliefs about the intervention and self-efficacy

A consistent challenge identified by dietitians was the need to rely on their clinical judgement and adapt the pathway to suit patient’s needs when high quality clinical evidence was not available. Over time, the dietitians became more comfortable with using their professional judgement to adapt the pathway, particularly where the evidence base was weaker.

#### Process

##### Engagement

Along with the previous cited perceptions about improved communication, the MDT survey respondents also identified significant benefits to seeing the dietitian before surgery (Table 2).

##### Execution

The barriers identified in the previous CFIR constructs made the execution of the pathway challenging, and sometimes impossible to achieve despite the dietitian’s best efforts (Table 2).

##### Reflection

Throughout the study, dietitians reflected on their role coordinating the nutrition appointments during each patient’s ever-changing and complex clinical journey and that the barriers were mainly surrounding coordination of care. By the end of the study there were reflections on the improvements in patient care as a result of the pathway including being able to provide more proactive care, improved relationships with the MDT and having a greater understanding of the patient journey.

### Construct relationships

**Fig. 2:**
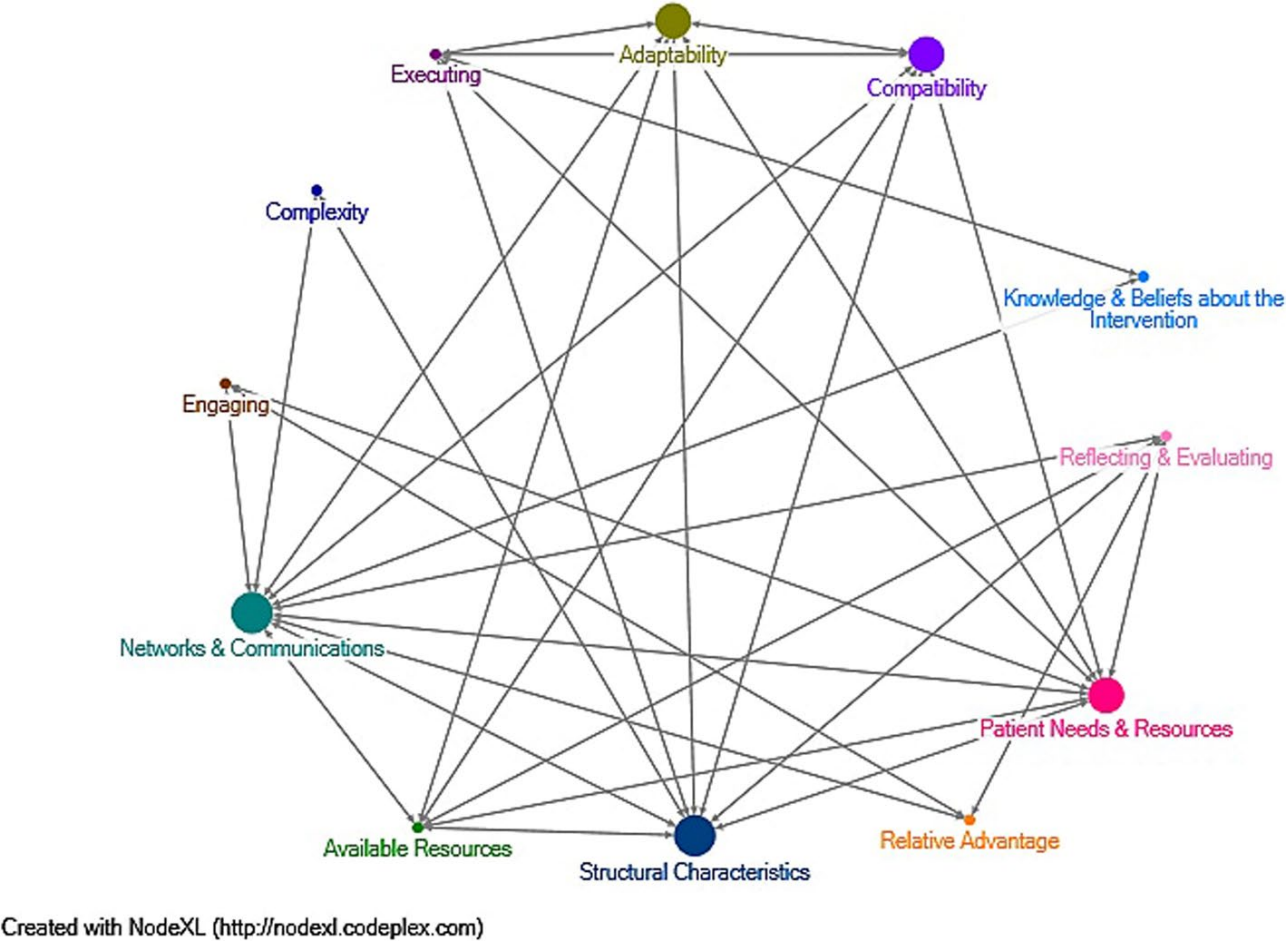
Relationships between Consolidated Framework for Implementation Research constructs discussed by dietitians during focus groups

The inner setting was the most prominent domain in the implementation experience discussed in the dietitian focus groups, with regards to networks and communication, and structural characteristics. The data related to these constructs overlapped significantly and were also closely related to patient needs and resources, in turn affecting adaptability and compatibility of the pathway. Figure 2 demonstrates the interaction between the CFIR constructs discussed by dietitians in the focus groups, circles size representing prominence of construct discussion. Figure 2 was created by Dedoose qualitative analysis software (SocioCultural Research Consultants, LLC, 2019, Los Angeles, CA). Dedoose identifies code co-occurrences by mapping all code pairings that are applied to the same or overlapping excerpts and displaying them in a matrix. The size of the circles correlates to the number of times this construct was identified in the data, while the lines represent identified co-occurrences between them.

## Discussion

Understanding the successes and challenges posed by implementing interventions in order to effectively translate knowledge into practice is becoming increasingly recognised in oncology, particularly if the intervention involves a complex care pathway [16]. This is the first study to evaluate nutrition care pathway implementation in cancer patients, using a validated framework. Other studies have described the compliance post implementation or assessed adherence to the intervention [17–19], but have not utilised an implementation framework for multidimensional analysis. The main benefits arising from pathway implementation in this study included increased engagement and communication between dietitians and the MDT and a more proactive approach, resulting in high overall levels of satisfaction of nutrition care from all stakeholders. Challenges to implementation largely involved issues related to pathway adaption to make it compatible with other aspects of medical care, which was influenced by communications, structural characteristics and patient needs. The significant overlap between the key constructs as outlined in the CFIR analysis demonstrates the complex clinical and implementation environment in which the dietitians attempted to use the pathway.

Co-location of clinicians from various disciplines can promote multidisciplinary care within the outpatient clinic setting [20]. The nutrition care pathway model of care, particularly the inclusion of the dietitian in the weekly outpatient clinic, allowed for increased engagement and communication between the dietitian and the MDT. However individual patient variability and the need to adapt remained a consistent feature of the dietitian’s experiences throughout the study. Although the benefits to standardised care pathways have been demonstrated in other oncology populations [6], this study demonstrates that the complexity and heterogeneity within the UGI cancer patient population pose challenges to the standardisation of nutrition care; particularly as certain aspects lack robust evidence to support recommendations [4]. Clinicians using a nutrition pathway need to be aware of the strength of evidence that guides care and also have strong clinical judgement skills, which can be challenging for those new to the field. Care pathways can be beneficial as a guide to management for clinicians but may not be compatible with all patient situations. Other studies describing sustainability post implementation of a nutrition care pathway in haematology cancer patients have also reported the need to undertake ‘practical’ changes to the pathway post implementation, particularly where the evidence to support practice was weak or based on expert consensus [17].

Although the MDT outpatient clinic was viewed as the most optimal way to deliver care in this study, it was difficult for dietitians to execute the pathway when patients did not attend appointments as scheduled. Other studies have demonstrated low attendance to standalone outpatient models of supportive allied health care in cancer patients, due to reasons including patients being too unwell or not wanting to travel to appointments [20], as seen in this study. Considerations of the patient emotional wellbeing and preferences often played a role in the dietitian’s decisions to modify the pathway. Innovative approaches to improve flexibility towards patient needs and reduce utilisation of dietetics resources could be considered, such as pre-recorded education videos, telehealth programs, or increased utilisation of joint consultations with surgeons or oncologists. A randomized controlled trial investigating intensive dietetics intervention for UGI cancer patients via a mobile app is currently being undertaken [21], with findings potentially being translatable to be incorporated into a nutrition care pathway model of care.

Whilst utilisation of structured protocols and pathways have been reported as a significant enabler to the treatment of malnutrition in cancer patients [22], the pathway implemented in this study relied on the dietitian to drive the referrals and arrange follow up care. However, the coordination of appointments was not expected to be a significant aspect of their role prior to the study. It became evident that the pathway was not adequately integrated into existing clinical structures and processes, resulting in decreased compatibility in the inner setting. This was an unexpected finding from the study, although this confirms previous research that the context can largely impact on whether practice change is successfully achieved [23]. Although a dietitian is best placed to drive change to nutrition care, ideally the pathway should be implemented within the medical care pathway, and responsibility of implementation shared across the team. Williams et al. describe the implementation of a multi-disciplinary preoperative nutrition optimisation clinic (POET) and pathway in a recent publication [24]. Whilst the dietitian is the integral clinician for delivery of nutrition care, screening of patients and referral into the pathway are performed by a range of treating clinicians. The clinic therefore aims to focus dietetics resources to delivery of nutrition intervention as this is where specialist skills are most valuable, but also to increase awareness and responsibility for recognition of malnutrition across all disciplines involved in patient care. Furthermore, the barriers faced in this study with regards to care coordination may largely be due to the lack of adequate funding for nurse coordinators at the participating sites. In other multi-disciplinary surgical optimisation programs, including the POET clinic, nurse coordinators are deemed as an essential member of the team who facilitate coordination and communication across departments, and the patient [24, 25].

The systematisation of multi-disciplinary care in this context also requires further exploration, which may include strategies to ensure that all clinicians can view a patient’s progress in their treatment journey in ‘real time’. Findlay et al. successfully implemented an evidence-based model of nutrition care in head and neck cancer, by ensuring integration with the MDT using a live Nutrition Care Dashboard that was incorporated into the weekly multi-disciplinary meeting [26]. Nutrition information and handover was also standardised as part of the electronic medical record to ensure continuity of care between clinicians and care settings [26]. This approach could be beneficial to overcome barriers discussed in this study, particularly those related to communication and compatibility. However, the contextual complexities described in this study, including shared care between institutions and multimodal treatments, pose significantly different challenges to a head and neck cancer population receiving radiotherapy as a single treatment modality, at a single institution. The POET clinic also implemented an online dashboard and system of direct transfer of surgical notes to the dietitian to facilitate communication and collaboration, however it is noted that patients attend clinic and begin the nutrition pathway only once they are scheduled for surgery [24]. The nutrition pathway implemented in this study is unique as it spans the entire preoperative oncological treatment pathway as well as the immediate pre-surgical period. The pathway aims to provide nutrition care as early as possible which optimises patient care, but challenges are faced when care is being provided over an extended time period, and across multiple treatment stages. It is noted that the pilot period was relatively short in this study. As the pathway becomes more ingrained and established into practice over time, communication between members of the MDT and dietitian may improve.

### Strengths and limitations

Strengths of this study include the use of mixed methods to obtain data from a wide range of stakeholders including members of the MDT and patients, which were integrated in the final analysis. Dietitian focus group data was collected longitudinally throughout the study and on completion, reflecting the transpiring aspects of implementation across the study period. The intervention and implementation data collection were conducted across four sites, enabling a rich understanding of challenges and benefits across hospital settings. Limitations of the study include the fact that all data collected were self-reflections of a relatively small sample of size of participants, and differences between sites were not investigated. The site leads participated repeatedly in the focus groups, which could bias the perspectives, however other dietitians involved in the study also participated and during coding dominance from one person/site was not observed. Included quotes were selected to be as representative as possible. The study lead conducted the focus groups and analysed the data therefore bias may have been introduced in data collection, however this was minimised during analysis by having a second coder who was not involved in the project and did not know the participants. Furthermore, qualitative data from patients and the MDT was not collected, due to funding limitations.

## Conclusion

This study provides detailed insights regarding the implementation of a nutrition care pathway in a ‘real world’ clinical setting. Overall, the benefits to the pathway compared to standard care were well recognised by all participants, and the MDT outpatient clinic model of care enabled the most compatible environment for success of the pathway. However, challenges to successful implementation arising from complex clinical and structural environments resulted in a significant coordination burden for dietitians and a reduced ability to execute the pathway effectively. Findings suggest that for this nutrition care pathway to be successful it requires integration into MDT care. In addition, coordination and communication regarding the patient’s medical care requires improvement at a systems level. Further exploration of systematic integration nutrition care into standard treatment pathways is required.

## Data Availability

The data-sets generated and/or analysed during the current study are not publicly available due to ethical review restrictions, but aggregated, de-identified data are available from the corresponding author on reasonable request.

## Abbreviations

UGI: Upper gastrointestinal
MDT: Multi-disciplinary team
CFIR: Consolidated Framework for Implementation Research
PSCNS: Patient Satisfaction with Clinical Nutrition Services
